# An intergranular strain concept for material models formulated as rate equations

**DOI:** 10.1002/nag.3043

**Published:** 2020-02-06

**Authors:** Manuel Bode, Wolfgang Fellin, David Mašín, Gertraud Medicus, Alexander Ostermann

**Affiliations:** ^1^ Unit of Geotechnical and Tunnel Engineering University of Innsbruck Austria; ^2^ Faculty of Science Charles University Czech Republic 128 43 Prague; ^3^ Department of Mathematics University of Innsbruck Austria

**Keywords:** barodesy, hypoplasticity, small‐strain, reloading, soil cyclic behaviour

## Abstract

The intergranular strain concept was originally developed to capture the small‐strain behaviour of the soil with hypoplastic models. A change of the deformation direction leads to an increase of the material stiffness. To obtain elastic behaviour for smallstrains, only the elastic part of the material stiffness matrix is used. Two different approaches for an application of this concept to nonhypoplastic models are presented in this article. These approaches differ in the determination of the elastic stress response, which is used for reversible deformations. The first approach determines an elastic response from the original material model, and the second one uses an additional elastic model. Both approaches are applied on barodesy. The simulations are compared with experimental results and with simulations using hypoplastic models with the original intergranular strain concept.

## 1 INTRODUCTION

**Figure 1:**
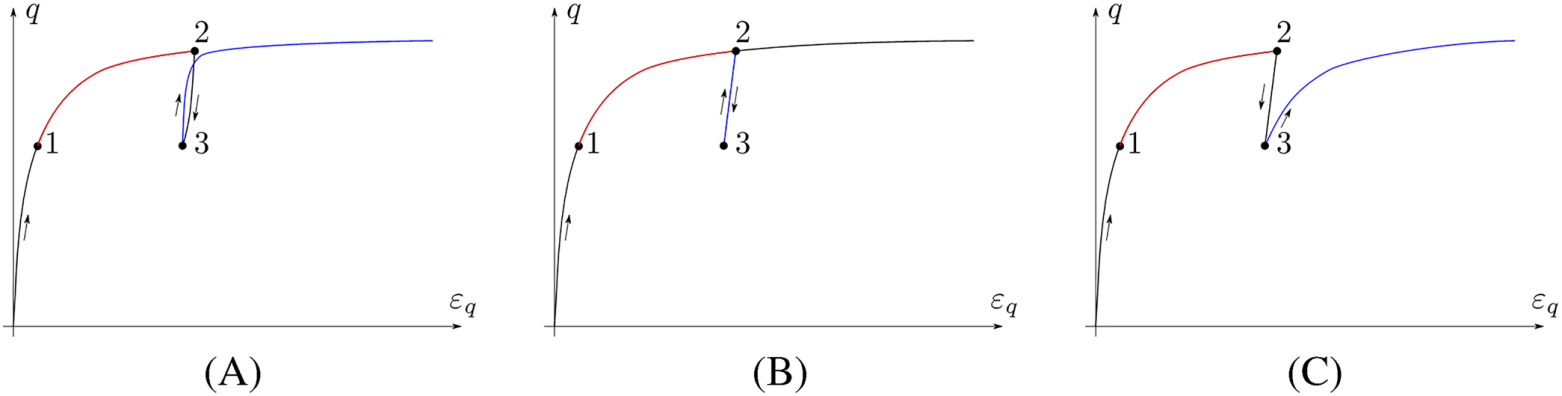
Loading and reloading in triaxial tests. It is assumed that at points 1 and 3 the same stress T, the same stretching D, and the same void ratio e occur. A, Realistic soil behaviour schematically. After the load reversal at point 3, the stiffness for reloading is increased compared with monotonic loading (1‐2). B, Basic elastoplastic models do not distinguish between unloading and reloading stiffness. C, Basic hypoplastic and barodetic models of the type $\overset{\circ }{T}=f(T,D,e)$ do not distinguish between monotonic loading and reloading and therefore underestimate the reloading stiffness [Colour figure can be viewed at http://wileyonlinelibrary.com]

The development of material models is a major field of research in soil mechanics. One specific issue is modelling the behaviour of soil in cyclic loading, ie, the deformation of soil, which is loaded, unloaded, and reloaded several times. The reloading behaviour differs significantly from the behaviour at first loading. Additionally, any reloading does not simply follow the reverse unloading path. We outline this peculiar behaviour qualitatively in Figure 1, starting with the reloading behaviour in triaxial tests in Figure 1A. The difficulties of the majority of constitutive relations to model cyclic loading are already revealed at the first reloading: with basic elastoplastic models, reloading is purely elastic, so all subsequent cycles of loading and unloading do not cause any additional irreversible deformation (Figure 1B). With basic hypoplastic models^1^, ^2^ and barodesy,^3^, ^4^ however, it proves difficult to distinguish the initial loading from subsequent reloadings, and hence too large irreversible deformations are predicted. An accumulation of deformation, the so‐called ratcheting effect, is obtained in small stress cycles (Figure 1C). The problem related to reloading is inherently linked with the so‐called small‐strain stiffness.

### 1.1 The role of memory in barodesy and hypoplasticity

Barodesy and hypoplasticity store the memory in the state variables T (stress) and e (void ratio). This proves to be a good concept for monotonic loading. Without further information, however, a distinction between first loading and reloading is not possible. This shortage is negligible in many cases, but it proves deleterious for reloading and for so‐called small‐strain problems.^5^, ^6^

**Figure 2:**
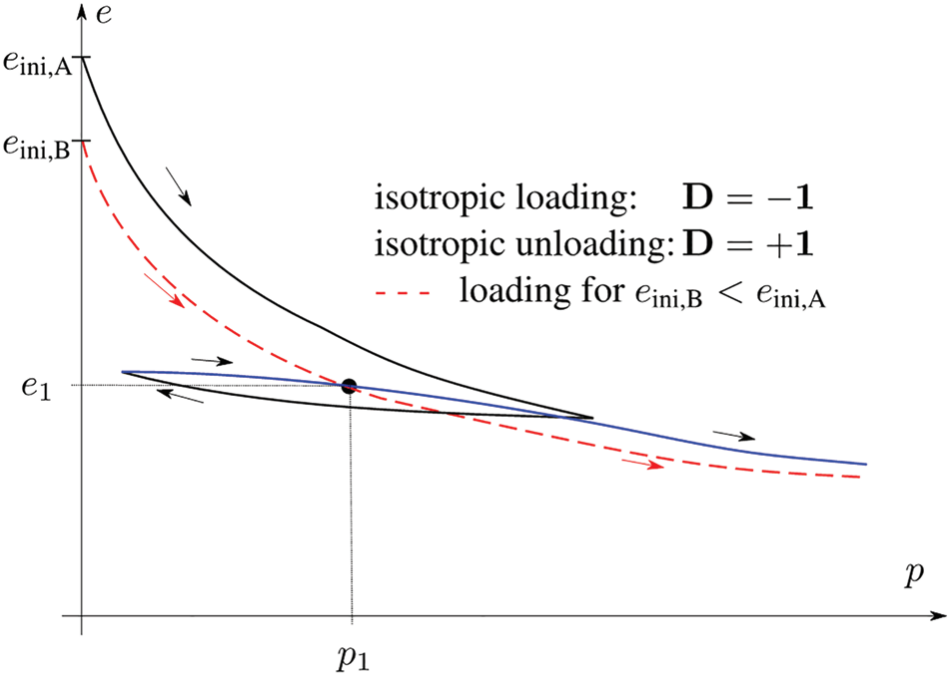
The role of memory in soil behaviour (schematically): Isotropic compression of soil. For (cyclic) reloading, the stiffness at $p_{1}$ is increased compared with that for monotonic loading (dashed line) passing through the void ratio $e_{1}$ . Thus, the void ratio $e_{1}$ and the stress $p_{1}$ are not sufficient to describe both, monotonic loading and the reloading behaviour. Figure slightly modified from Herle^5^ [Colour figure can be viewed at http://wileyonlinelibrary.com]

In Figure 2, the isotropic compression behaviour of soil is shown schematically. For (cyclic) reloading, the stiffness at $p_{1}$ is strongly increased compared with that for monotonic loading passing through the same void ratio $e_{1}$ (dashed line). Thus, the void ratio $e_{1}$ and the stress $p_{1}$ are not sufficient to describe both monotonic loading and reloading behaviours. The same conclusion can be drawn if we consider triaxial compression of soil; see Figure 1C. After the load reversal at point 3, the stiffness for reloading is increased compared with that for initial loading (1‐2). It is assumed that at points 1 and 3, the same stress T, the same stretching D, and the same void ratio e occur. Again, the void ratio e and the stress T are not sufficient to describe both, initial loading and the reloading behaviour.

To improve the *memory* of the soil model, one needs to consider what the independent variables in a constitutive relation of the rate type are. If a model does not include any additional information about the deformation history, it has to be enhanced with special extensions to capture the small‐strain behaviour. For elastoplastic models, the *small strain overlay model* 6 is a possible approach. It uses a strain history surface as memory. To model small‐strain effects and thus to overcome the ratcheting effect in hypoplastic models, Niemunis and Herle^7^ introduced the *intergranular strain concept*. In this model, the so‐called intergranular strain is introduced as an additional state variable that acts as memory of former deformations.

This concept was extended by several authors, eg, Wegener and Herle,^8^ Mašín^9^ or Fuentes and Triantafyllidis,^10^ to improve its performance and to capture more soil features. Wegener and Herle^8^ added an additional material parameter to control the transition between the small strain and the large strain model. This parameter is used to reduce the predicted strain accumulation for cyclic loading. Mašín^9^ added an anisotropic model for the linear part of the material model and allowed for a nonlinear dependence of the very smallstrain shear modulus on the mean effective stress. Fuentes and Triantafyllidis 10 introduced the intergranular strain anisotropy (ISA) model, which incorporates a yield surface within the intergranular strain space. This allowed for improved predictions of the memory effects upon reloading paths, which also improved predictions of the strain accumulation in cyclic tests and reduced the overshooting effects. In its original form, the intergranular strain concept is designed for hypoplastic models. We propose two possible extensions of the intergranular strain concept to make it applicable to other material models of the rate type. For simplicity, we only use the basic intergranular strain concept as introduced by Niemunis and Herle^7^ without any modifications; however, the above‐mentioned improved versions can also be used with our presented approaches. In the last section, we give a brief outlook about a possible application of the improved versions. We only consider models that are positively homogeneous of degree one in the stretching D, ie, rate‐independent models, although the original model could also be used for models considering rate effects as the visco‐hypoplastic model.^11^

**Figure 3:**
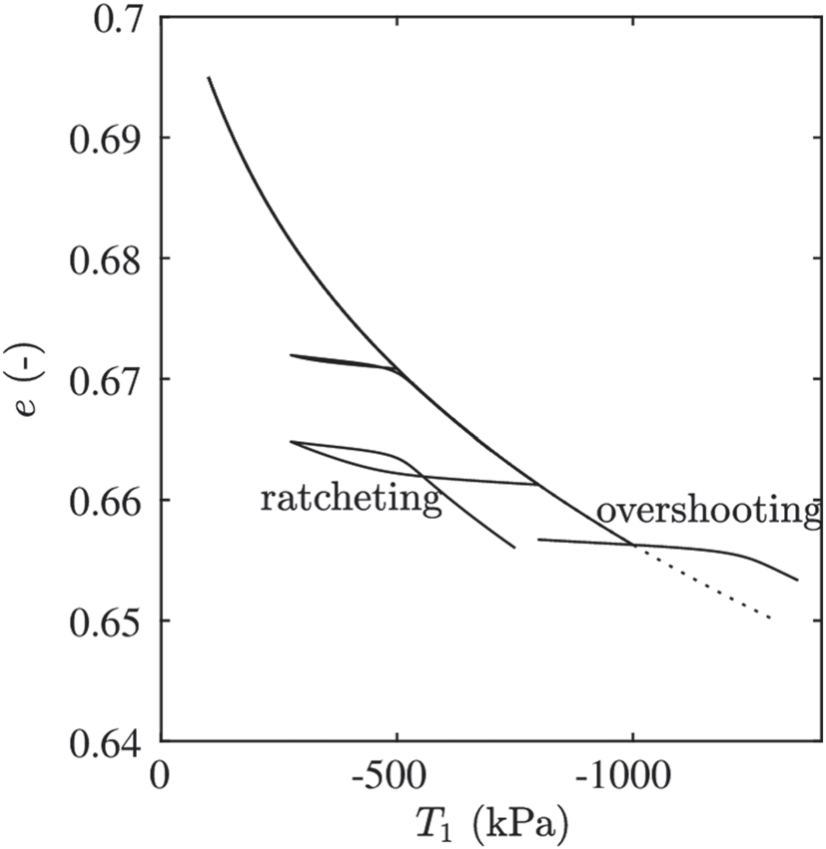
Simulated oedometric compression tests using hypoplasticity^16^ show some shortcomings of the intergranular strain concept for different reloading cycles. Parameters of Hochstetten sand^7^ starting at isotropic stress state with $p_{0}=100\text{kPa}$

The good performance and acceptance of the intergranular strain model is shown by a wide range of applications.^12^, ^13^, ^14^ However, the original intergranular strain concept also has some shortcomings; eg, for different load cycles, using the same set of parameters, effects of ratcheting, and overshooting can appear. These effects are shown in Figure 3 for simulations of different load cycles in oedometric compression tests using hypoplasticity with the original intergranular strain concept. As the concept uses a hypoelastic model where a complete reversibility of stress upon a closed strain cycle is not ensured, the concept is not suitable for high‐cyclic problems. Wichtmann et al^15^ recently compared the behaviour of three sophisticated models for cyclic loading, including the intergranular strain concept and the ISA model. For all models, they showed advantages and shortcomings under different conditions; eg, hypoplastic models using the intergranular strain concept perform better than the ISA model for the accumulation rate under stress‐ and strain‐controlled undrained cycles, whereas the opposite is true for cyclic mobility effects.

The approaches presented here do not represent an improvement of the original intergranular strain concept. They will serve as an application of the intergranular strain concept to other models, which opens the possibility to improve their small‐strain behaviour. Moreover, making the intergranular strain concept accessible for a wider range of constitutive models can be a motivation to create new developments and to finally improve the concept itself.

### 1.2 Symbols and notation

In this article, stress is denoted by T and stretching by D. Compression is defined as negative. Second‐order tensors are written in bold capital letters (eg, X), $\|X\|=\sqrt{\text{tr}\;X^{2}}$ is the Euclidean norm of a symmetric tensor X, and trX is the sum of the diagonal components of X. The superscript 0 marks a normalized tensor, ie, $X^{0}=X/\|X\|$. Any symmetric second‐order tensor can be written as vector with the principal values $X_{v}=\left[X_{1},X_{2},X_{3}\right]$. We use this to display tensors in figures; however, we do not use the notation $X_{v}$, as it is implicitly clear that X is shown as a vector in these figures. Bold calligraphic letters denote tensors of the fourth order (eg, M). We use different kinds of tensor operations using the Einstein summation convention. In particular, the indices follow the lexicographic order $X⊗Y=X_{ij}Y_{kl}$, $X:Y=X_{ij}Y_{ij}$, and $L:D=L_{ijkl}D_{kl}$. We employ the unit tensor of second order I with $I_{ij}=\delta_{ij}$ and the fourth‐order tensor I with $I_{ijkl}=\delta_{ik}\delta_{jl}$ using the Kronecker delta $\delta_{ij}$.

Stresses are considered as effective ones; the normally used dash is omitted. The objective stress rate is denoted by $\overset{\circ }{T}$. The stretching tensor D is the symmetric part of the velocity gradient. The void ratio e is the ratio of the volume of the voids $V_{p}$ to the volume of the solids $V_{s}$. The additional state variables of the material models are collected in the vector Q. The effective mean stress is denoted by $p=-\frac{1}{3}\text{tr}\;T$. For a conventional triaxial compression or an oedometric compression test, the axial stress is denoted with $T_{1}$ and the radial stress is denoted with $T_{2}=T_{3}$. The associated strains are $\varepsilon_{1}$ and $\varepsilon_{2}=\varepsilon_{3}$. Deviatoric stresses and strains are denoted by $q=-(T_{1}-T_{3})$ and $\varepsilon_{q}=-\frac{2}{3}(\varepsilon_{1}-\varepsilon_{3})$.

## 2 INTERGRANULAR STRAIN CONCEPT

Before we present our approaches for the extension of the intergranular strain concept, we shortly outline the original concept and investigate some features that are useful for understanding our proposed extensions. For a detailed derivation of the basic concept, we refer to the original publication.^7^ We start with a general formulation of hypoplasticity

(1) $$\overset{\circ }{T}=f(T,D,Q)=L(T,Q):D+N(T,Q)\|D\|.$$

In this formulation, the terms of the hypoplastic equation are collected in terms linear in D, ie, (L:D), and terms that are not linear in D, ie, N‖D‖. In hypoplasticity, the tensor N does not depend on D, and the use of ‖D‖ leads to a nonlinear behaviour.^17^

To model a stiffness increase subsequent to a change in deformation direction, an additional tensorial state variable, the so‐called intergranular strain tensor δ, is introduced as memory of former deformations. We need the normalized tensor

(2) $$\delta^{0}=\frac{\delta }{\left\|\delta \right\|},$$

which can be interpreted as direction in the principal stretching space and the magnitude

(3) $$\rho =\frac{\left\|\delta \right\|}{R}$$

of the intergranular strain normalized with the maximum intergranular strain magnitude R. The intergranular strain tensor δ follows an evolution equation

(4) $$\overset{\circ }{\delta }=\left\{\begin{array}{ll} \left(I-\rho^{\beta_{r}}\delta^{0}⊗\delta^{0}\right):D & \;\text{for}\;\delta^{0}:D>0,\\ D & \;\text{for}\;\delta^{0}:D\leq 0, \end{array}\right.$$

which must be integrated together with the evolution Equation (1) of the stress rate. Here, R and $\beta_{r}$ are material parameters.

The stress rate is given by

(5) $$\overset{\circ }{T}=M:D,$$

with the material stiffness matrix

(6) $$M=[\rho^{\chi }m_{T}+(1-\rho^{\chi })m_{R}]L+\left\{\begin{array}{cc} \rho^{\chi }(1-m_{T})\left(L:\delta^{0}\right)⊗\delta^{0}+\rho^{\chi }N⊗\delta^{0} & \;\text{for}\;\delta^{0}:D>0,\\ \rho^{\chi }(m_{R}-m_{T})\left(L:\delta^{0}\right)⊗\delta^{0} & \;\text{for}\;\delta^{0}:D\leq 0. \end{array}\right.$$

The scalar variables $m_{T}$, $m_{R}$, and χ are material parameters, so, in total, five additional material parameters are needed. For a monotonic deformation, the maximum intergranular strain is reached, hence ρ=1. A sharp change of the deformation direction following a continuous monotonic deformation yields the maximum increase of stiffness. In case of a full reversal with $D^{0}=-\delta^{0}$, the material behaviour is elastic, as only the linear part of the stiffness matrix is used, which is increased by a scalar factor $m_{R}$. For an orthogonal change of direction with $D^{0}⊥\delta^{0}$ (or $\delta^{0}:D^{0}=0$) and ρ=1, Equation (5) with Equation (6) reduces to $\overset{\circ }{T}=m_{T}L:D$. For monotonic continuation of deformation with $D^{0}=\delta^{0}$ (or $\delta^{0}:D^{0}=1$) and ρ=1, we get the original material model as given in Equation (1). The scalar factor $\rho^{\chi }$ is used to perform an interpolation with change of the magnitude of δ.

**Figure 4:**
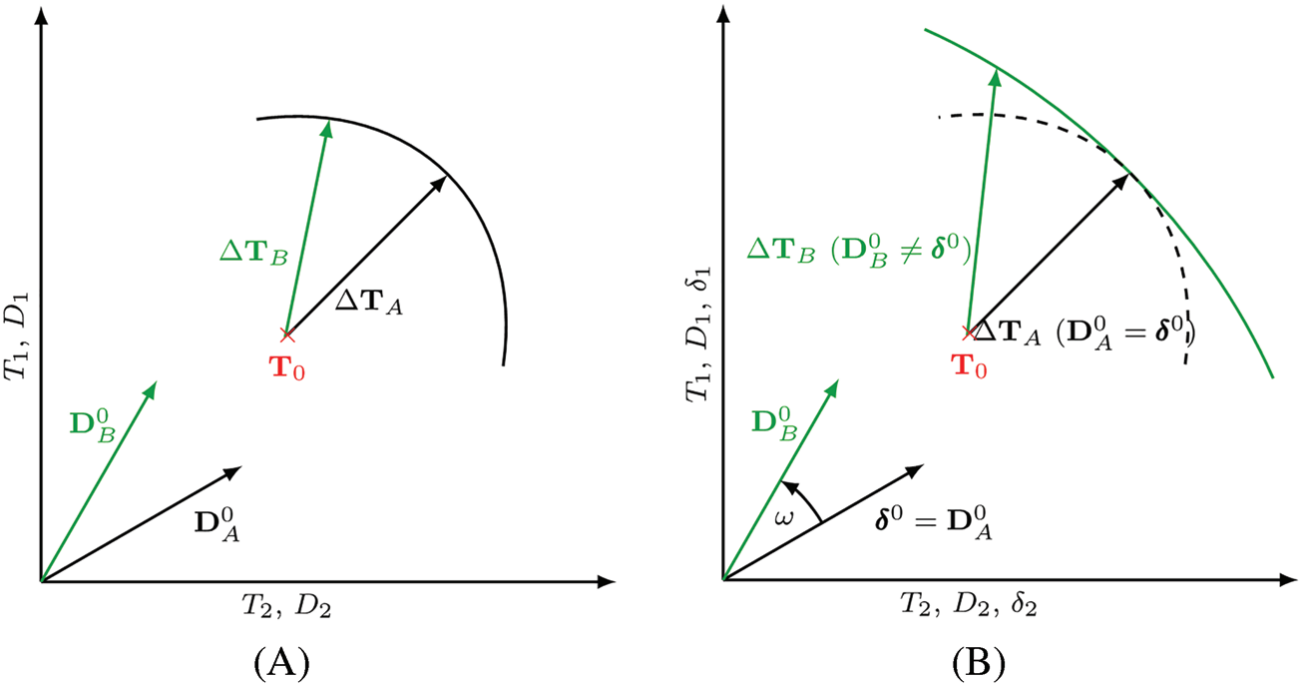
A, Resulting stress increments ΔT for given deformation rates D starting at the initial stress state $T_{0}$. B, For different directions of $D^{0}$ and $\delta^{0}$– differing by the angle ω – the stiffness gets increased compared with the original stiffness (dashed line) [Colour figure can be viewed at http://wileyonlinelibrary.com]

A useful graphical representation to visualize the directional response of a constitutive model are response envelopes.^18^ The stress response ΔT for the stretching D is visualized by multiplying the stress rate $\overset{\circ }{T}$ with a single time increment Δt as a scaling factor. The distance between the initial state $T_{0}$ and the point on the response envelope is a measure of the incremental stiffness; see Figure 4A. In Figure 4B, the intergranular strain concept is used. The directions of $D_{A}^{0}$ and $\delta^{0}$ are the same, ie, the stiffness is the same compared with that of the original material model. The direction of $D_{B}^{0}$ differs from $\delta^{0}$, and thus the stiffness is increased.

**Figure 5:**
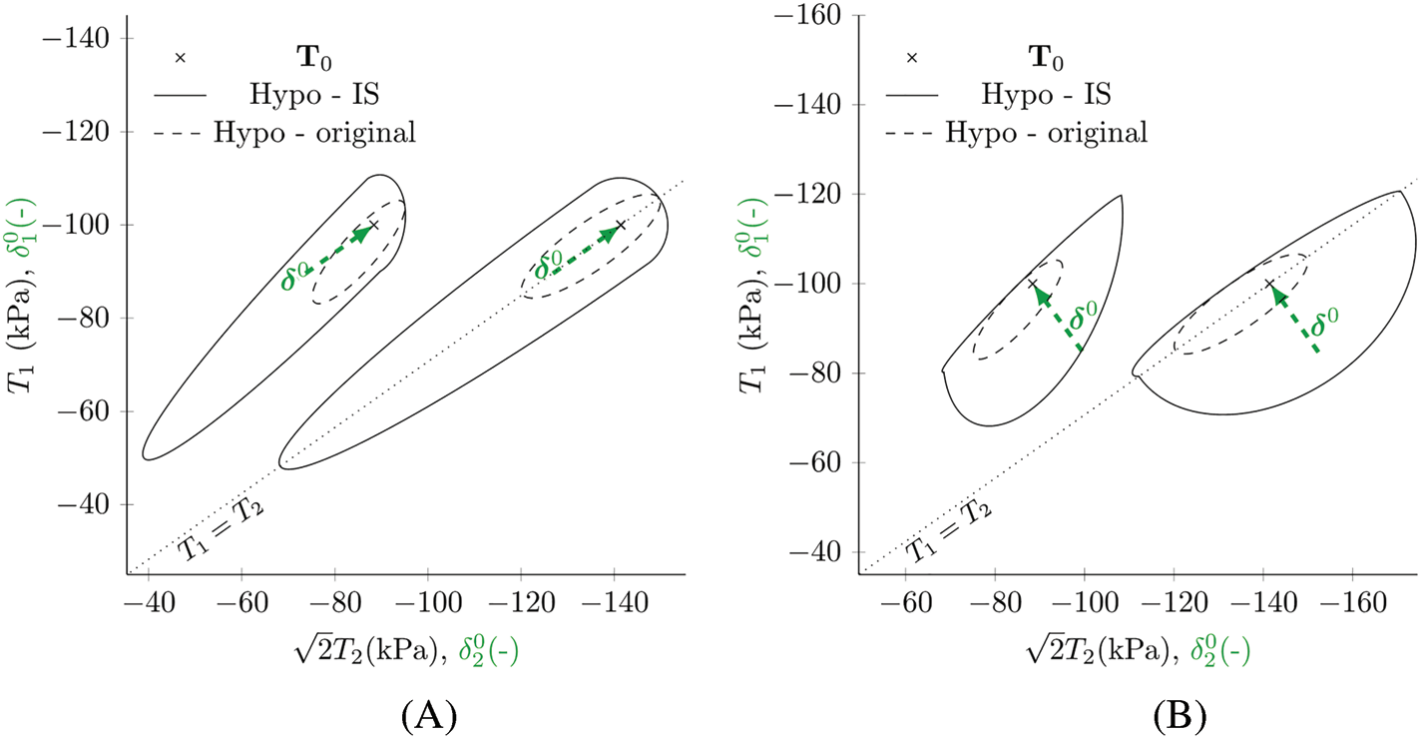
Response envelopes for hypoplasticity in the version of von Wolffersdorff with and without intergranular strain (IS) for different initial stress states $T_{0}$ and different former deformations with ρ=1. The arrows show the direction of intergranular strain $\delta^{0}$ [Colour figure can be viewed at http://wileyonlinelibrary.com]

Figure 5 shows the results for response envelopes simulated with hypoplasticity in the version of von Wolffersdorff^16^ using the material parameters for Hostun sand given by Niemunis and Herle.^7^ The arrows show the former deformation direction, which is stored in the intergranular strain tensor $\delta^{0}$. The response envelopes for hypoplastic models are ellipses,^18^ the shape of which is given by the linear part L. The centre of the ellipse is shifted from the initial stress point by the nonlinear part N. A response envelope with its centre at the initial stress point maps elastic behaviour, as the stiffnesses in reversal directions are equal. As small‐strain behaviour is assumed to be elastic, for reversal directions ( $\delta^{0}:D^{0}\leq 0$), only the part linear in D is used and scaled by $m_{R}$ and $m_{T}$, respectively. For directions with $\delta^{0}:D^{0}>0$, the nonlinear part is faded in, to obtain the original material behaviour.

As it is needed for further discussion, we combine Equations (5) and (6),

(7) $$\overset{\circ }{T}=f_{A}\underset{A}{\underbrace{L:D}}+\left\{\begin{array}{cc} f_{B1}\underset{B}{\underbrace{\left(L:\delta^{0}\right)⊗\delta^{0}:D}}+\rho^{\chi }\underset{C}{\underbrace{N⊗\delta^{0}:D}} & \;\text{for}\;\delta^{0}:D>0,\\ f_{B2}\underset{B}{\underbrace{\left(L:\delta^{0}\right)⊗\delta^{0}:D}} & \;\text{for}\;\delta^{0}:D\leq 0, \end{array}\right.$$

with the following abbreviations:

(8) $$\begin{array}{ll} f_{A} & =[\rho^{\chi }m_{T}+(1-\rho^{\chi })m_{R}],\\ f_{B1} & =\rho^{\chi }(1-m_{T}),\\ f_{B2} & =\rho^{\chi }(m_{R}-m_{T}). \end{array}$$

Equation (7) depends on three different tensors A,B, and C. The expression $\delta^{0}:D^{0}=\cos\omega$ serves as interpolation function to consider different angles ω between the direction of intergranular strain $\delta^{0}$ and the direction of actual deformation $D^{0}$; see Figure 4B. With $\eta =\delta^{0}:D^{0}=\cos\omega$, and $D=\|D\|D^{0}$, we can write

(9) $$\delta^{0}:D=\eta \|D\|.$$

We get η=1 for monotonic deformation with $\delta^{0}=D^{0}$ and η=0 for perpendicular deformation with $\delta^{0}⊥D^{0}$. For reverse of deformation, η=−1, and the second line in Equation (7) is used.

The concept of intergranular strain is designed for hypoplastic models. Its original formulation is strongly based on the structure of hypoplastic models, which consist of terms linear and nonlinear in D as shown in Equation (1). The part linear in D is used to calculate the elastic stiffness within the small strain range. For some constitutive models of the rate type

(10) $$\overset{\circ }{T}=f(T,D,Q),$$

this linear part cannot be explicitly extracted from the model formulation.

To be able to apply the intergranular strain concept to such material models without an explicit linear part, we need to offer a consistent elastic stress response for small strains. This is the motivation for creating a more general approach for an intergranular strain extension that can easily be used with other constitutive models of the rate type.

## 3 BARODESY

We use barodesy to show the application of our intergranular strain approaches. Barodesy, introduced by Kolymbas,^19^ is a material model that has similarities to hypoplasticity. As in hypoplasticity, the stress rate is formulated as a function of stress, stretching, and void ratio: $\overset{\circ }{T}=f(T,D,e)$. Barodesy^4^ also includes concepts from critical state soil mechanics as a stress‐dependent critical void ratio. In barodesy, critical stress states almost coincide with predictions by Matsuoka–Nakai.^20^ Compared with elastoplastic models, barodesy and hypoplasticity do not distinguish between elastic and plastic strain. In contrast to hypoplasticity, barodesy cannot be directly split into parts that are linear and nonlinear in D. The full set of equations for barodesy for clay can be found in the Appendix 1.

## 4 INTERNAL ELASTIC MODEL

The intergranular response is assumed to be elastic.^7^ In hypoplasticity, this is modelled with the linear part of the material model. In a more general approach, we need an appropriate stress rate that delivers equal stress rates for reversal stretchings

(11) $${\overset{\circ }{T}}_{\text{el}}(T,D,Q)=-{\overset{\circ }{T}}_{\text{el}}(T,-D,Q),$$

ie, ${\overset{\circ }{T}}_{\text{el}}$ is an odd function with respect to D. We denote by ${\overset{\circ }{T}}_{\text{el}}$ the incrementally elastic stress rate. The stress rate T can be decomposed in an incrementally elastic part ${\overset{\circ }{T}}_{\text{el}}$ and a nonelastic part ${\overset{\circ }{T}}_{\text{ne}}$ with

(12) $$\overset{\circ }{T}={\overset{\circ }{T}}_{\text{el}}+{\overset{\circ }{T}}_{\text{ne}}.$$

The incrementally elastic stress rate ${\overset{\circ }{T}}_{\text{el}}$ is the odd part of $\overset{\circ }{T}$ with respect to D. In the case of hypoplasticity, ${\overset{\circ }{T}}_{\text{el}}$ coincides with L:D, which is the part of Equation (1) that is linear in D. Note, however, that for more complex material models, ${\overset{\circ }{T}}_{\text{el}}$ is not necessarily linear in D. In our first approach, the given material model is directly used to compute the elastic stress rate. The resulting model will be called *internal elastic model* (IEM).

### 4.1 Decomposition of the material model

In Equation (7), the three tensors A, B, and C were identified, where evaluations of the elastic and the nonelastic parts of the constitutive model are necessary. The incrementally elastic stress rate in Equation (11) represents the odd part of the constitutive Equation (10) with respect to the stretching D. This part can be obtained by evaluation of the constitutive Equation (10) once with the actual stretching D and once with the reverse direction −D:

(13) $$\begin{array}{ll} {\overset{\circ }{T}}^{+} & =f(T,D,Q),\\ {\overset{\circ }{T}}^{-} & =f(T,-D,Q). \end{array}$$

The odd part is then obtained as

(14) $${\overset{\circ }{T}}_{\text{el}}=\frac{1}{2}\left({\overset{\circ }{T}}^{+}-{\overset{\circ }{T}}^{-}\right).$$

The rest of the stress rate in direction of D is the nonelastic part, which is also the even part of the constitutive relation with respect to D:

(15) $${\overset{\circ }{T}}_{\text{ne}}=\overset{\circ }{T}-{\overset{\circ }{T}}_{\text{el}}=\frac{1}{2}\left({\overset{\circ }{T}}^{+}+{\overset{\circ }{T}}^{-}\right).$$

The incrementally elastic part of the stress rate ${\overset{\circ }{T}}_{\text{el}}$ is the first expression A in Equation (7). In expression C, the nonelastic part of the material model ${\overset{\circ }{T}}_{\text{ne}}$ is used.

Expression B in Equation (7) considers different directions of the actual deformation and the intergranular strain, to perform a smooth transition between the tangential and the reversal stiffness increase. For hypoplasticity, B is the evaluation of the linear part of the constitutive model in the direction of the intergranular strain. For our formulation, the odd part of the constitutive equation in direction of intergranular strain is used. Setting $D=\delta^{0}$ in Equation (10) and denoting ${\overset{\circ }{T}}_{\delta }=f(T,\delta^{0},Q)$, we can use the same procedure as before:

(16) $$\begin{array}{ll} {\overset{\circ }{T}}_{\delta }^{+} & =f(T,\delta^{0},Q),\\ {\overset{\circ }{T}}_{\delta }^{-} & =f(T,-\delta^{0},Q), \end{array}$$

and further

(17) $${\overset{\circ }{T}}_{\delta \text{el}}=\frac{1}{2}\left({\overset{\circ }{T}}_{\delta }^{+}-{\overset{\circ }{T}}_{\delta }^{-}\right).$$

This finally provides a possible extension of the intergranular strain concept within the IEM approach. Employing the abbreviations in Equations (8) and (9), we get

(18) $$\overset{\circ }{T}=f_{A}{\overset{\circ }{T}}_{\text{el}}+\left\{\begin{array}{cc} f_{B1}\eta_{1}{\overset{\circ }{T}}_{\delta \text{el}}\|D\|+\rho^{\chi }\eta_{1}{\overset{\circ }{T}}_{\text{ne}} & \;\text{for}\;\delta^{0}:D>0,\\ f_{B2}\eta_{2}{\overset{\circ }{T}}_{\delta \text{el}}\|D\| & \;\text{for}\;\delta^{0}:D\leq 0. \end{array}\right.$$

Note that we are using two different interpolation functions $\eta_{1}$ and $\eta_{2}$ for more flexibility. Both functions depend on the variable $\omega =\arccos(\delta^{0}:D^{0})$. At $\omega =90^{\circ }$, ie, $\delta^{0}:D^{0}=0$, they must vanish to ensure a smooth transition between the two cases in Equation (18). These functions are $\eta_{1}=\eta_{2}=\eta$ for hypoplasticity; see Equation (9).

Applying these ideas to the hypoplasticity given in Equation (1), we get the expressions

(19) $$\begin{array}{ll} {\overset{\circ }{T}}_{\text{el}} & =L:D,\\ {\overset{\circ }{T}}_{\text{ne}} & =N\|D\|,\\ {\overset{\circ }{T}}_{\delta \text{el}} & =L:\delta^{0}. \end{array}$$

For hypoplasticity, the new concept thus reduces to the original intergranular strain concept.

The IEM approach models an increase of stiffness and an elastic response in a small‐strain range for a given constitutive model using the incrementally elastic part of the model. For constant stretching, Equation (18) leads to the response of the original constitutive model. We need four evaluations of the material model at each time step and each integration point to calculate the different parts of the model.

### 4.2 Application of the IEM approach to barodesy

We apply the IEM approach on barodesy for clay as an example of its use.^4^ As the IEM approach uses the behaviour of the given material model to calculate an elastic response and to increase the material stiffness, it also reproduces all its peculiarities, which must be considered to reproduce a realistic behaviour.

**Figure 6:**
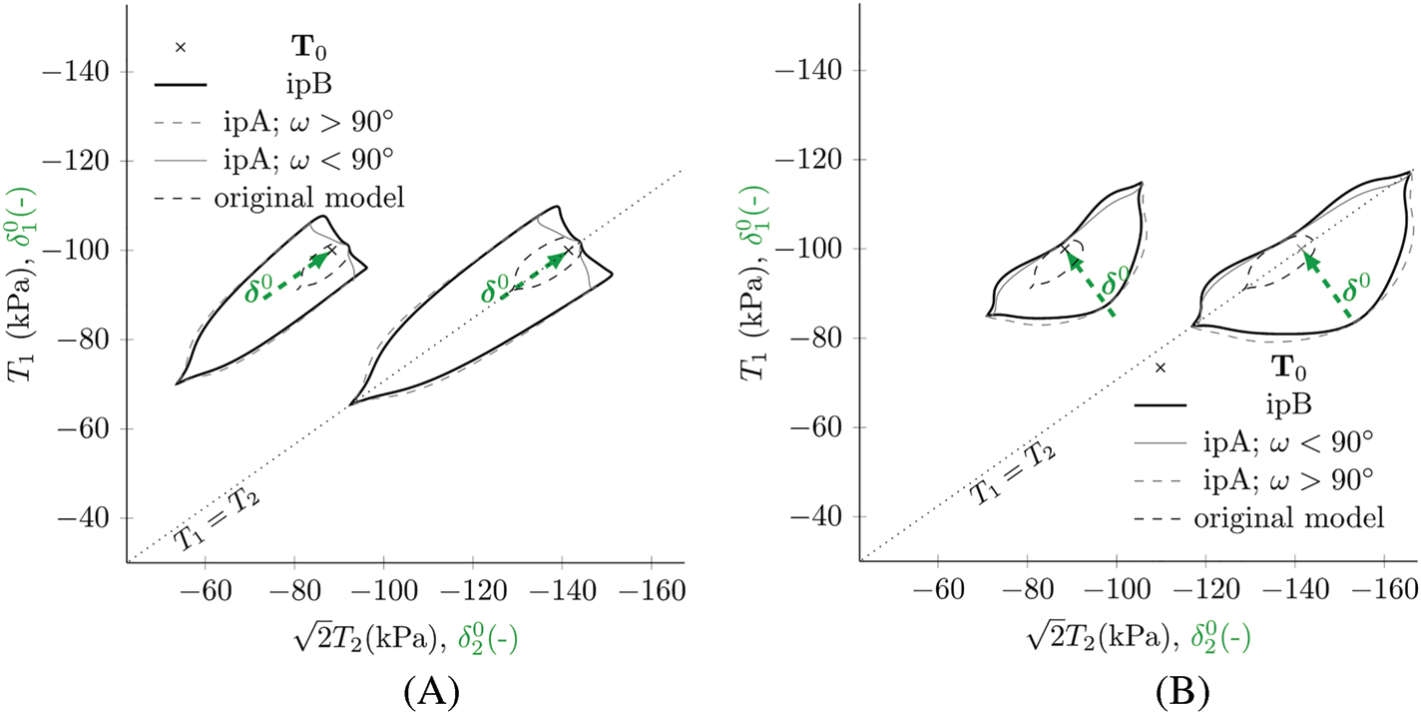
Response envelopes using barodesy with the parameters in Table 1 and the internal elastic model (IEM) approach for different initial stress states $T_{0}$ and different former deformations with ρ=1. ipA: $\eta_{1}=\eta_{2}=\cos\omega$; ipB: $\eta_{1}=-\eta_{2}=\cos^{2}\omega$. The arrows show the intergranular strain direction $\delta^{0}$ [Colour figure can be viewed at http://wileyonlinelibrary.com]

**Figure 7:**
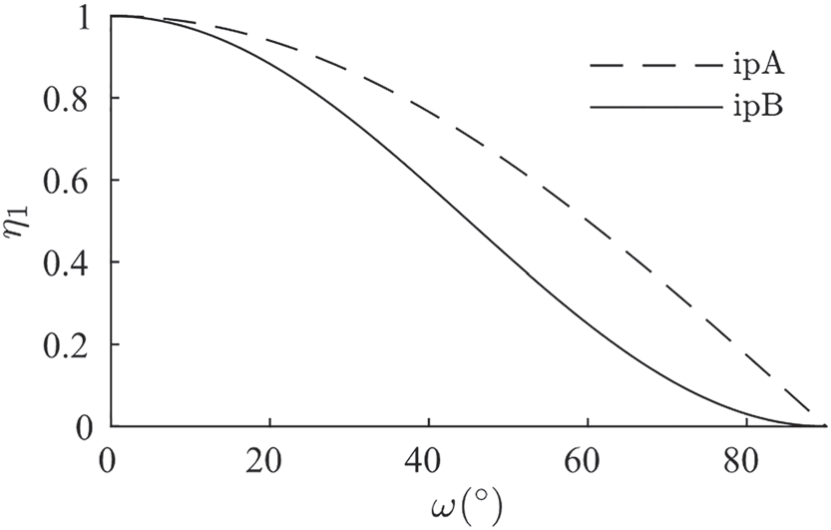
Different interpolation functions $\eta_{1}$ and $\eta_{2}$ used for Equation (18) and the simulations in Figure 6. ipA: $\eta_{1}=\eta_{2}=\cos\omega$; ipB: $\eta_{1}=-\eta_{2}=\cos^{2}\omega$

The intergranular strain concept using the IEM approach can be regarded as a blow‐up of the original response envelope. Thus, the original shape strongly influences the resulting one. The elliptical shape of response envelopes in hypoplasticity is only an assumption, as the exact shape is not known from experiments.^22^, ^23^ Response envelopes of barodesy are roughly elliptical but not smooth for isotropic unloading; see Figure 6. Through the interpolation process, this irregularity can also appear in other parts of the intergranular strain response envelope, where the original response envelope is smooth. This is a result of the directional interpolation of the parts ${\overset{\circ }{T}}_{\delta \text{el}}$ and ${\overset{\circ }{T}}_{\text{ne}}$ with $\eta_{1}$ for $\delta^{0}:D>0$ in Equation (18). The intergranular strain response with $\eta_{1}=\eta_{2}=\cos\omega$ for $\omega >90^{\circ }$ as shown in Figure 6 is not realistic, because a small change of the direction $D^{0}$ to the direction $\delta^{0}$ leads to a decrease of stiffness. Henceforth, we call this interpolation scheme *ipA*. A simple way to adjust the directional stress response is a change in the interpolation function η. In our case, we want to get horizontal tangents in the interpolation functions $\eta_{1}$ and $\eta_{2}$ for $\omega =0^{\circ }$ and $\omega =90^{\circ }$ to reach a smooth transition between the two cases in Equation (18). This can be done by using

(20) $$\eta_{1}=\frac{\left(1+\cos2\omega \right)}{2}=\cos^{2}\omega$$

and $\eta_{2}=-\eta_{1}$. The horizontal tangent for $\omega =90^{\circ }$, as shown in in Figure 7, now leads to a smooth transition between the parts of the model. This interpolation is denoted by *ipB*.

**Table 1:** Default parameters employed for barodesy (left) and the intergranular strain extensions (right), used for the evaluation of the models. These parameters are fictitious values, only used for the evaluation of the models^21^

$\varphi_{c}$	N	$\lambda^{*}$	$\kappa^{*}$	$m_{R}$	$m_{T}$	R	$\beta_{r}$	χ
$25^{\circ }$	1	0.1	0.01	4.5	2.25	$10^{-4}$	0.2	6

Using the *ipB* interpolation scheme, we get a more realistic shape of the intergranular strain response envelope for barodesy, which is shown by the black solid lines in Figure 6.

For other material models, other interpolation functions $\eta_{1}$ and $\eta_{2}$ may be more suitable. This can be investigated by simulation of response envelopes for different δ and different initial stress states.

## 5 EXTERNAL ELASTIC MODEL

Another approach to get an elastic response for intergranular strain is the following: We define an external elastic model (EEM) and perform an interpolation between the original constitutive model and the external model using the framework of the original intergranular strain concept. Here, as an example, we use an elastic model with the stiffness matrix

(21) $$M^{e}=2G\left(I+\frac{\nu }{1-2\nu }I⊗I\right),$$

with the elastic shear modulus G and the Poisson ratio ν.

We start with the original intergranular strain concept given in Equation (7). The model response should be elastic within the small‐strain range. For hypoplasticity, the elastic response is achieved with the part linear in D. We replace the linear part L with the elastic stiffness matrix $M^{e}$. Hence, for a reversal of the deformation, only the elastic model is used and scaled up with the factor $m_{R}$. With ongoing constant stretching, the enlarged stress rate has to reduce to the stress rate of the original model, denoted by ${\overset{\circ }{T}}_{m}$. We can thus replace the nonlinear part N‖D‖ with the stress rate of the constitutive model ${\overset{\circ }{T}}_{m}$ minus the stress rate resulting from the elastic model. A smooth transition between the different models is guaranteed by using the interpolation framework of the original intergranular strain concept. With these assumptions, we get a formulation for the objective stress rate $\overset{\circ }{T}$ employing the abbreviations from Equation (8) and the interpolation function $\eta =\eta_{1}=\eta_{2}=\cos\omega$:

(22) $$\overset{\circ }{T}=f_{A}M^{e}:D+\left\{\begin{array}{cc} f_{B1}\eta \|D\|M^{e}:\delta^{0}+\rho^{\chi }\eta \cdot \left({\overset{\circ }{T}}_{m}-(M^{e}:D)\right) & \;\text{for}\;\delta^{0}:D^{0}>0,\\ f_{B2}\eta \|D\|M^{e}:\delta^{0} & \;\text{for}\;\delta^{0}:D^{0}\leq 0. \end{array}\right.$$

### 5.1 Determination of the elastic model

The main task in this approach is determining the elastic model parameters – in this case, G and ν from Equation (21) – which should be consistent with the original model, and finding a proper calibration. Only if the elastic model is consistent with the original model, a proper interpolation and calibration of the intergranular strain parameters is possible.

We can determine an incremental bulk modulus K and a shear modulus G from the original material model by calculating the model response for isotropic and isochoric deformation. For this hypoelastic model, we use the assumption that the intergranular strain behaviour is independent from the stress ratio, so we can perform these calculations at an isotropic stress state.

Calculating the stress rate for isotropic loading, the incremental bulk modulus can be obtained as

(23) $$K_{\text{il}}=\frac{\dot{p}}{\text{tr}\;D},$$

with the time rate of the effective mean stress $\dot{p}$ and the volumetric stretching trD. The same can be done for isotropic unloading, resulting in $K_{\text{iu}}$. We take then $K=\frac{1}{2}(K_{\text{il}}+K_{\text{iu}})$, which is an average value of incremental loading and unloading stiffness from the original model.

The shear modulus G links the deviatoric strain with the deviatoric stress and can be estimated as stiffness for deformations with constant volume. We need to calculate the stiffness for an isochoric deformation, ie, trD=0. For an isotropic material, the shear stiffness is the same in all (isochoric) deviatoric directions. Thus, one calculation is enough to assess the shear modulus, as it can be adjusted by calibration of the stiffness parameters $m_{R}$ and $m_{T}$ to fit the initial small‐strain shear stiffness $G_{0}$. For a calculation under axially symmetric conditions and in principal stresses, we get

(24) $$2G=\frac{\dot{T_{1}}-\dot{T_{3}}}{D_{1}-D_{3}}.$$

The elastic stiffness matrix can be written as

(25) $$M^{e}=2GI+\left(K-\frac{2}{3}G\right)I⊗I.$$

Note that Equations (21) and (25) are the same with $K=\frac{2G(1+\nu )}{3(1-2\nu )}$.

In the general case, we need three evaluations of the material model to get the elastic parameters and one evaluation to get the material response considering intergranular strain at the actual state. So again, we need four evaluations of the material model at each time step and each integration point, if the parameters cannot be extracted analytically from the model formulation.

### 5.2 Calibration of the EEM model

For a full reversal of the deformation direction, the stiffness should be increased by the scalar factor $m_{R}$. For a calibration of the stiffness increase, the pressure‐dependent small‐strain shear stiffness $G_{0}$ is used, which is known from laboratory tests, eg, Mašín^24^ or estimated by empirical relations, eg, Hardin and Black^25^ and Biarez and Hicher.^26^

Using the formulation of the elastic stiffness matrix from Equation (21) with $\nu =\frac{3K-2G}{6K+2G}$, the calibration procedure presented by Mašín^21^ for hypoplasticity is applicable. We get

(26) $$M^{e}=2GL$$

with

(27) $$L=I+\frac{\nu }{1-2\nu }I⊗I.$$

After a full reversal of deformation direction with ρ=1, Equation (22) yields

(28) $$\overset{\circ }{T}=m_{R}M^{e}:D.$$

With Equation (26),

(29) $$\overset{\circ }{T}=2G_{0}L:D=2m_{R}G\cdot L:D,$$

we get

(30) $$m_{R}=\frac{G}{G_{0}}.$$

For the calibration of $m_{R}$, we just need to calculate the incremental elastic shear modulus G for the same pressure, for which the small‐strain shear modulus $G_{0}$ is known.

### 5.3 Application of the EEM approach to barodesy

We apply the EEM approach to barodesy for clay as an example. By predefining the respective boundary conditions, we can directly calculate the incremental elastic parameters at an isotropic state for barodesy. This reduces computational effort as not the entire determination of the elastic parameters has to be done at each time step.

As for hypoplasticity, the response envelope of barodesy at the isotropic state does not change its shape by changing the overconsolidation ratio (OCR). A different OCR only leads to a shift of the centre of the response envelope away from the initial stress point $T_{0}$. In barodesy, the incremental shear modulus and the average bulk modulus stay the same for a different OCR. At normally consolidated isotropic states in barodesy, the bulk modulus for isotropic loading is directly given with the parameter $\lambda^{*}$ as

(31) $$K_{\text{il}}=p\frac{1}{\lambda^{*}}=pK_{\text{il}}^{*}$$

with $K_{il}^{*}=\frac{1}{\lambda^{*}}$. For isotropic unloading, we use Equation (A1) inserting $T^{0}=R^{0}=-\frac{1}{\sqrt{3}}I$ and $\text{tr}\;D^{0}=\sqrt{3}$. The stress rate for isotropic unloading

(32) $$\overset{\circ }{T}=p\left(2c_{3}\left(2^{\lambda^{*}c_{5}}-1\right)-\frac{\sqrt{3}}{\kappa^{*}}\right)\frac{{\dot{\varepsilon }}_{\text{vol}}}{\sqrt{3}}I$$

is only dependent on the actual mean pressure p and the volumetric strain rate for isotropic unloading with ${\dot{\varepsilon }}_{\text{vol}}=\sqrt{3}\|D\|$. The parameters $c_{3}$ and $c_{4}$ are constants that can be calculated from the critical state material parameters; see Appendix 1. We thus get the bulk modulus for isotropic unloading with $\overset{\circ }{T}=-\dot{p}\cdot I$.

(33) $$K_{\text{iu}}=\frac{\dot{p}}{{\dot{\varepsilon }}_{\text{vol}}}=p\left(\frac{1}{\kappa^{*}}-\frac{2c_{3}}{\sqrt{3}}\left(2^{\lambda^{*}c_{5}}-1\right)\right)=pK_{\text{iu}}^{*}.$$

Using the average value $K^{*}=\frac{1}{2}(K_{\text{il}}^{*}+K_{\text{iu}}^{*})$ for the linear elastic model (25), we get

(34) $$K^{*}=\frac{1}{2}\left(\frac{1}{\kappa^{*}}-\frac{2c_{3}}{\sqrt{3}}\left(2^{\lambda^{*}c_{5}}-1\right)+\frac{1}{\lambda^{*}}\right).$$

The incremental average bulk modulus $K=pK^{*}$ only depends on the actual mean pressure. The shear modulus G can be calculated with an isochoric deformation $D_{11}=-2D_{22}$, $D_{22}=D_{33}$ (here, $\text{tr}\;D^{0}=0$) and Equation (24):

(35) $$G=\frac{h\cdot \left(f\cdot \left(R_{11}^{0}-R_{22}^{0}\right)+g\cdot \left(T_{11}^{0}-T_{22}^{0}\right)\|D\|\right)}{3D_{11}}.$$

The definitions of the functions h(‖T‖), $f(D^{0})$, $g(D^{0},p)$ and $R(D^{0})$ are given in Appendix 1. At the isotropic state $T_{11}^{0}=T_{22}^{0}$ and for isochoric compression with $D_{11}<0$, we get $\|D\|=-\sqrt{\frac{2}{3}}D_{11}$. The shear modulus for isochoric compression starting at the isotropic state is obtained with $h=c_{3}\sqrt{3}p$ as

(36) $$G=p\frac{c_{3}}{2\sqrt{2}}\left(R_{11}^{0}-R_{22}^{0}\right)=pG^{*}$$

and

(37) $$G^{*}=\frac{c_{3}}{2\sqrt{2}}\left(R_{11}^{0}-R_{22}^{0}\right).$$

The shear and bulk moduli for barodesy for clay at the isotropic state only depend on the actual mean pressure. The stiffness coefficients $K^{*}$ and $G^{*}$ only depend on material parameters and can be used to calculate the linear elastic material stiffness matrix. Thus, Poisson ratio here is also independent of the pressure and can be calculated as

(38) $$\nu =\frac{3K-2G}{6K+2G}=\frac{3K^{*}-2G^{*}}{6K^{*}+2G^{*}}.$$

Using this calibration, we obtain a hypoelastic model with a pressure‐dependent shear modulus and a constant Poisson ratio. The stiffness matrix according to Equation (26) reads

(39) $$M^{e}=f_{e}2G^{*}L$$

with the stiffness factor $f_{e}=p$.

**Figure 8:**
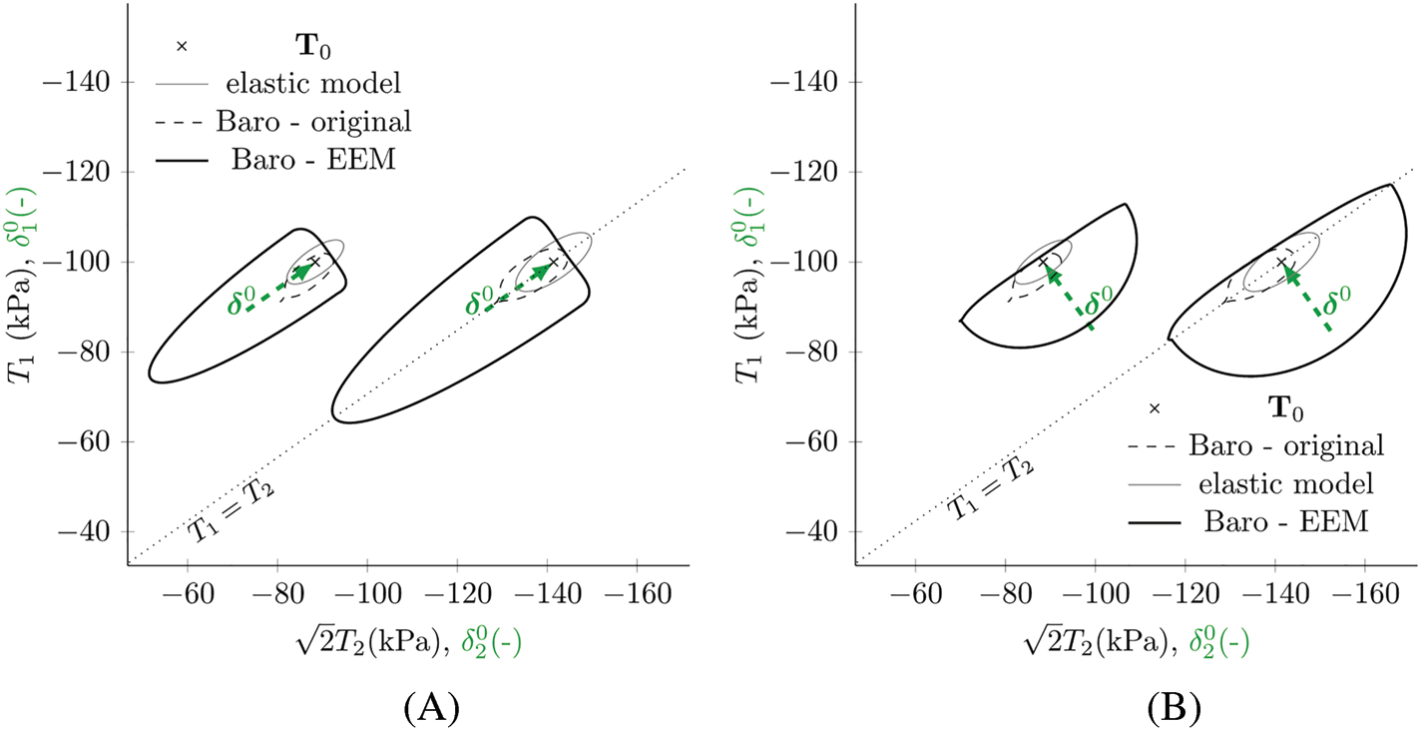
Response envelopes using barodesy with the parameters in Table 1 and the external elastic model (EEM) approach for different initial stress states $T_{0}$ and different former deformations with ρ=1. The arrows show the intergranular strain direction $\delta^{0}$. The initial states are the same as for the simulations with the internal elastic model (IEM) approach in Figure 6 [Colour figure can be viewed at http://wileyonlinelibrary.com]

Figure 8 shows response envelopes simulated with barodesy using the EEM approach. The dashed lines show the original response envelopes and the grey solid lines the responses from the linear elastic model centred at the initial stress state. The intergranular strain response is a combination of both model responses using the interpolation in Equation (22).

## 6 COMPARISON AND SIMULATIONS

In this section, we show and discuss some simulation results using both proposed approaches in combination with hypoplasticity in the versions of von Wolffersdorff (v.W.) and Mašín^21^ (clay hypoplasticity) as well as with barodesy. Those results are compared with experimental data and with simulations using the original intergranular strain concept.

### 6.1 Simulation results

**Figure 9:**
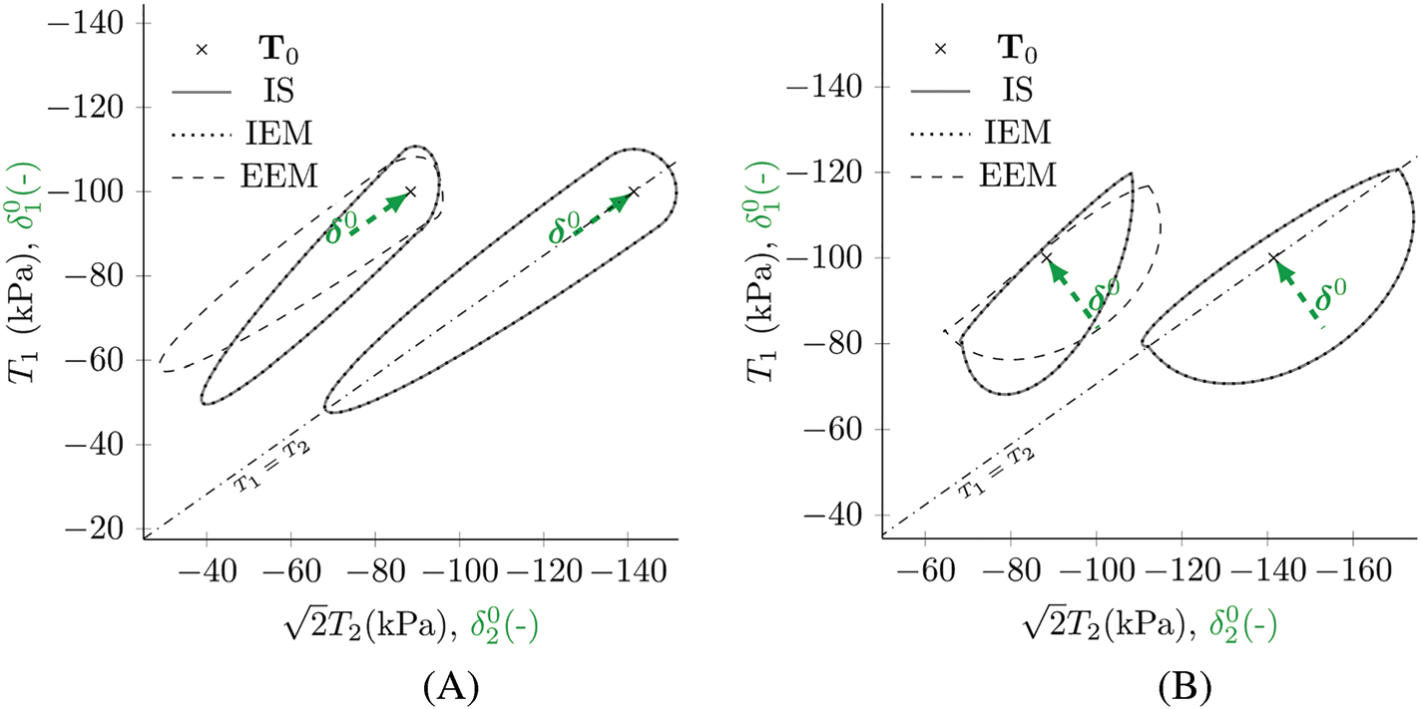
Response envelopes for von Wolffersdorff (v.W.) hypoplasticity using the proposed intergranular strain (IS) approaches. The initial states are the same as for the simulations in Figure 5. The arrows show the intergranular strain direction $\delta^{0}$. EEM, external elastic model; IEM, internal elastic model [Colour figure can be viewed at http://wileyonlinelibrary.com]

In a first step, we apply both approaches to hypoplasticity. The response envelopes using v.W. hypoplasticity for different intergranular strain directions and different initial stress states are shown in Figure 9. The IEM approach in combination with hypoplasticity yields exactly the same results as the original intergranular strain concept. The linear part in the v.W. hypoplastic model is dependent on the actual stress state, while the elastic model used for the EEM only depends on the mean stress and is independent of the deviatoric stress. This leads to different orientations of the response envelopes for nonisotropic stress states. In clay hypoplasticity, the linear part is also not effected by the deviatoric stresses.^21^ Combining this model with the IEM or the EEM approach leads to the same results as with the original intergranular strain concept.

We use triaxial tests on reconstituted London clay from Mašín,^24^ namely, PhM17, PhM19, and PhM21, for a comparison of the simulations with experimental data. A discussion of these tests is done by Mašín.^27^ We use barodesy for clay and clay hypoplasticity for the simulations. The employed parameters for London clay are given in Table 2. The boundary conditions for the simulations are shown in Table 3. The simulations start with a reverse intergranular strain direction.

**Table 2:** Material parameters for London clay^27^

Model	$\varphi_{c}$	N	$\lambda^{*}$	$\kappa^{*}$	ν
Barodesy	$22.6^{\circ }$	1.375	0.11	0.016	‐
Hypoplasticity	$22.6^{\circ }$	1.375	0.11	0.016	0.25

. The parameter ν for hypoplasticity is calculated from parameter r, as shown by Mašín.^21^

**Table 3:** Boundary conditions for the simulated triaxial tests^24^

Test	$\sigma_{a}^{\prime }$, kPa	$\sigma_{r}^{\prime }$, kPa	$e_{0}$	Conditions
PhM17	266	165	1.039	Constant p, extension
PhM19	100	115	1.113	Constant p, compression
PhM21	450	450	0.950	Undrained, compression

For the simulations with hypoplasticity, we use $m_{R}=4.5$. For the EEM approach, $G_{0}^{*}=\frac{G_{0}}{p}$ can be assessed using linear regression as shown by Mašín.^27^ We get $m_{R,\text{EEM}}=4.0$ for barodesy using Equations (37) and (30) to fit the hypoplastic calibration of test PhM19.^27^ As the IEM approach uses the response of the actual material model, the parameters $m_{R}$ and $m_{T}$ may differ from those of the EEM approach. We use $m_{R,\text{IEM}}=4.3$ for barodesy to get an equal stiffness for test PhM19. For all simulations, $m_{T}=0.5m_{R}$. The other intergranular strain parameters are the default values from Mašín,^27^ which are $\beta_{r}=0.2$, $R=10^{-4}$ and χ=6.

**Figure 10:**
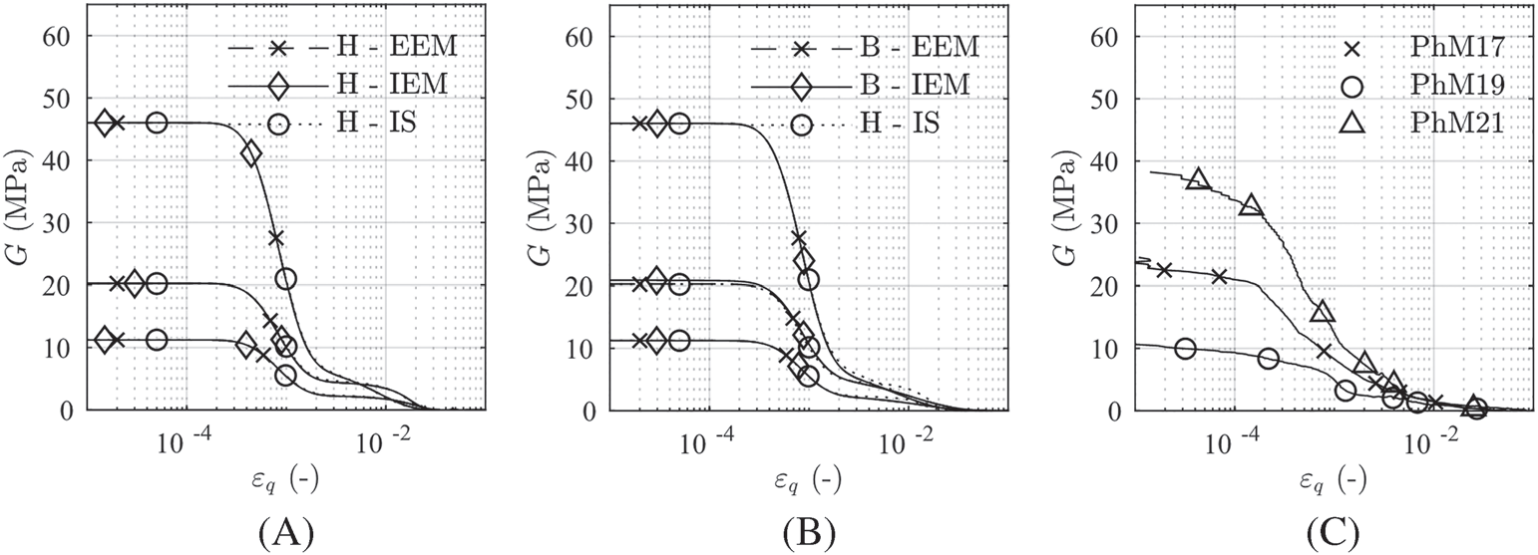
Predicted shear stiffness degradation for (A) clay hypoplasticity, H, and (B) barodesy, B, using the proposed approaches (external elastic model [EEM] and internal elastic model [IEM]); A, and B, also contain hypoplasticity using the original intergranular strain concept (IS); C, comparison with experimental data from Mašín^24^

Figure 10 shows the degradation of the shear stiffness for the tests PhM17, PhM19, and PhM21. As the shear stiffness is calibrated for test PhM19, all models show the same results within the small‐strain range. Using clay hypoplasticity, the proposed approaches show the same results as the original intergranular strain concept (Figure 10A.) With a change of the pressure and the stress states, the results of barodesy differ slightly. For test PhM21, the initial pressure of 450kPa was quite different to the pressure of 110kPa used for the calibration with test PhM19. According to Mašín,^27^ this may explain the difference between the simulations and the experimental results. Overall, however, with both approaches, barodesy is able to model those pressure effects and represent the experimental results in the same quality as hypoplasticity.

**Figure 11:**
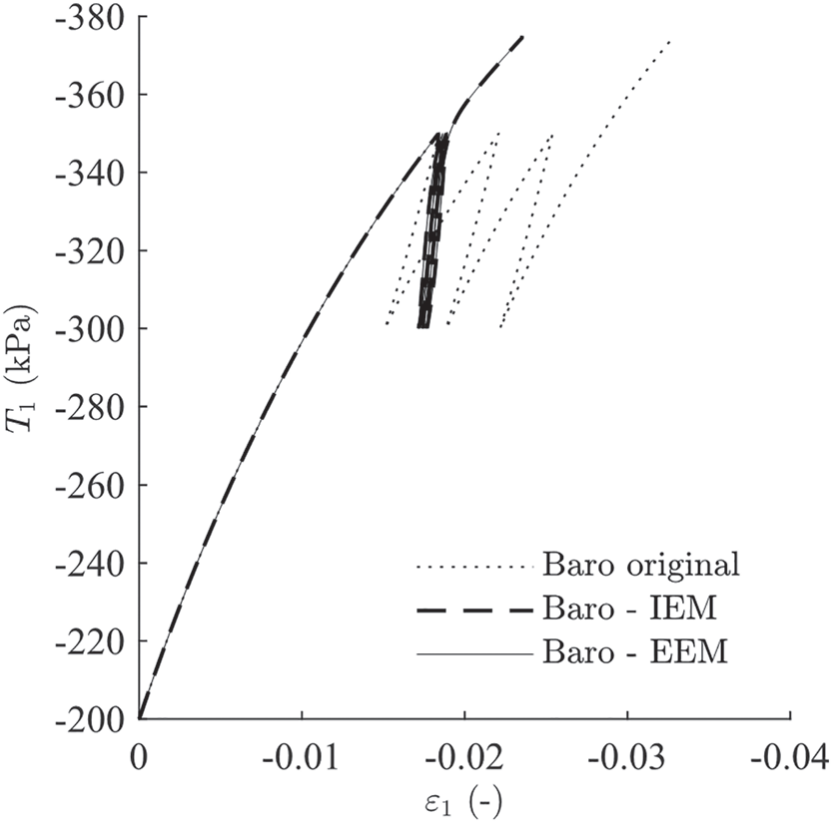
Undrained triaxial test with cyclic loading using parameters from Table 1 and starting with OCR=3. EEM, external elastic model; IEM, internal elastic model

**Figure 12:**
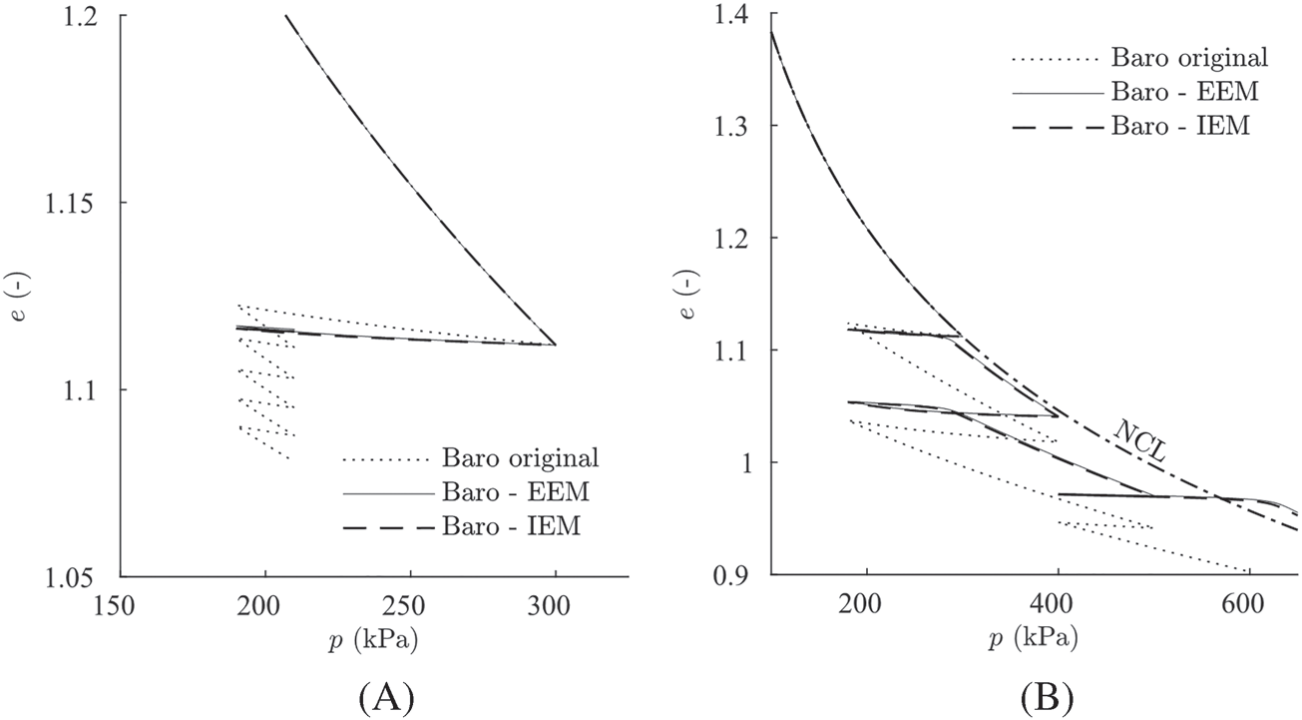
Simulations of normal consolidated isotropic compression tests with barodesy using parameters for London clay. A, Cyclic reloading; B, different reloading loops. EEM, external elastic model; IEM, internal elastic model; NCL, normal compression line

Standard laboratory tests are simulated using the proposed extensions of the intergranular strain concept shown in Figures 11 and 12. Both approaches, IEM and EEM, show almost the same results and present a clear improvement compared with the original material model, as the reloading stiffness is increased. The shortcomings of the original intergranular strain formulation also emerge by applying our approaches. The second and the third reloading loops for the isotropic compression test in Figure 12B show ratcheting and overshooting effects, respectively.

### 6.2 Application to the modified Cam‐Clay model

The proposed concepts can also be used for elastoplastic models. Basic elastoplastic models with isotropic hardening, such as the modified Cam‐Clay model^28^ (MCCM), behave elastically for unloading and reloading. Such models do not show ratcheting effects as hypoplastic or barodetic models, but they fail to model the hysteretic behaviour that occurs due to reloading, as shown in Figure 2. The here proposed small‐strain extensions yield an increase of the stiffness for small strains and the hysteretic loops as shown in Figure 1.

**Figure 13:**
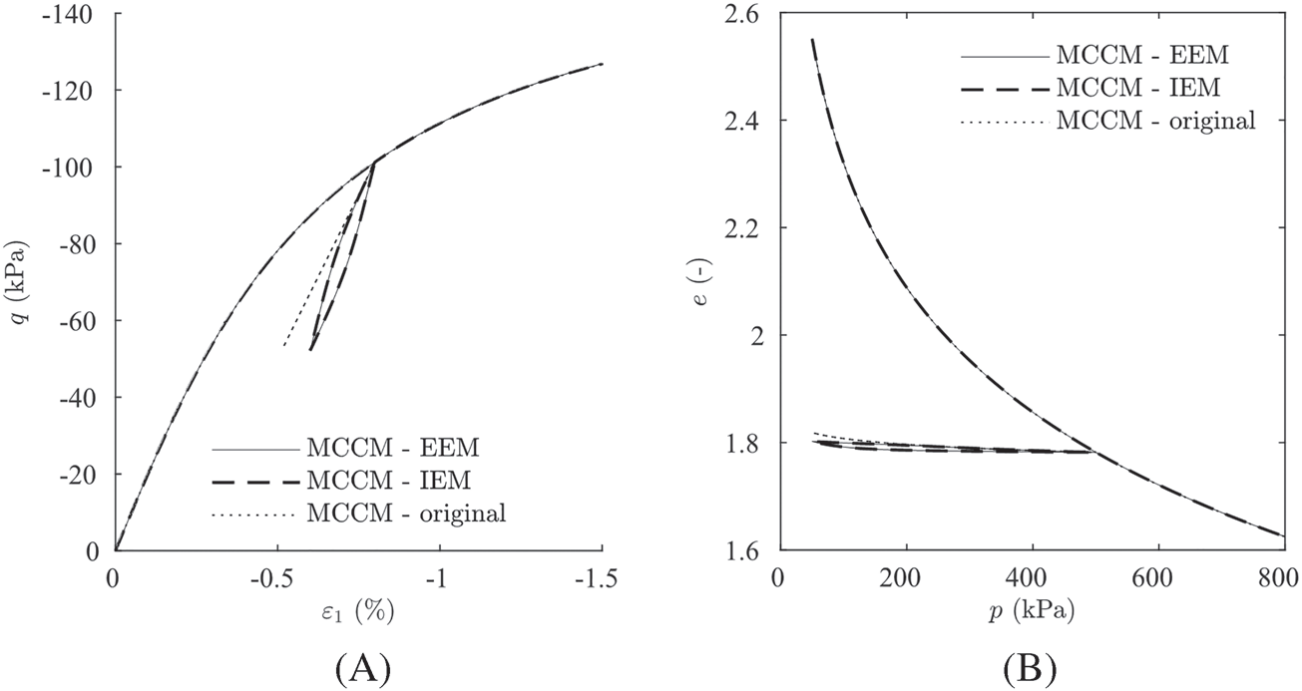
Simulations using the modified Cam‐Clay model (MCCM). (A) Normally consolidated undrained triaxial test with $p_{\text{ini}}=200\text{kPa}$. (B) Normal consolidated isotropic compression test. EEM, external elastic model; IEM, internal elastic model

The MCCM, enhanced with our intergranular strain approaches, is used for the simulations shown in Figure 13. The material parameters for MCCM are M=0.88, $v_{\lambda }=3.49$, λ=0.24, κ=0.044, and ν=0.3. The parameters for the elastic stiffness matrix for the EEM approach can be calculated as explained in Section 5.1 or directly be taken from the model parameters. Note that the methods to determine the elastic stiffness matrix lead to different results for states at the yield surface. At the yield surface, $K_{\text{il}}$ actually does not represent the elastic response. For the simulations in Figure 13, we directly use the material parameters of the MCCM and get $G=\frac{3K(1-2\nu )}{2(1+\nu )}$ with $K=v\frac{p}{\kappa }$ and $v=v_{\lambda }+\kappa \ln\frac{p_{0}}{p}$. For the normally consolidated simulations, it holds that $p_{0}=p_{\text{ini}}$. The parameters for the intergranular strain extension are set to the same values as for hypoplasticity. The IEM approach and the EEM approach show here virtually the same results.

### 6.3 General remarks

Small‐strain stiffness can be modelled with the IEM approach by increasing the elastic stiffness that is obtained using the original model. As an advantage of the IEM approach compared with the EEM approach, no additional model, including a calibration of additional model parameters, is necessary. The reproduced model behaviour is directly given by the original material model. As shown, however, using the original model response can lead to unrealistic responses because of the directional interpolation. This can be corrected by adjusting the interpolation function for a specific material model. For hypoplastic models using the formulation of Equation (1), the IEM approach leads exactly to the original intergranular strain concept; see Equation (19).

With the EEM approach, an additional external model is added, which is used to model the elastic behaviour in small‐strain range. This can be a simple elastic model, as presented in this article, but it can also be any other hypoelastic model.^7^ Using the EEM approach, it is possible to separate the small‐strain behaviour from the large‐strain model. For example, concepts of anisotropy as proposed by Mašín and Rott^29^ could easily be incorporated for the small‐strain range by changing the external model.

Both proposed approaches are able to use the basic intergranular strain concept as proposed by Niemunis and Herle^7^ and also any improved version. For an application of the approach of Wegener and Herle,^8^ to reduce the strain accumulation by changing the fade‐in of the nonlinear model, only the exponent χ in the third term of Equations (18) and (22) has to be changed. As the ISA model^10^ is also applicable to hypoplastic models using the L‐ N formulation,^30^ the possibility of applying the ISA model to other material models is provided by the approaches presented here. However, it has to be mentioned that the shortcomings of the original intergranular strain concept are also reproduced by the here presented approaches; see Figure 12.

Both approaches need in general four calls of the material model in each time step, so they need essentially the same computing time. The elastic parameters for the EEM approach can for some models directly be calculated, as shown for barodesy and the MCCM, so the same computational effort as for the original intergranular strain concept is needed.

## 7 SUMMARY

Two different approaches for extending the intergranular strain concept to nonhypoplastic models are presented in this article. The first approach – IEM – uses the properties of the original constitutive model and calculates an elastic response, which is used for the stiffness increase in the small‐strain range. For the second approach – EEM – an external elastic model is used for the calculation of the small‐strain stiffness, and an interpolation between the original model and the external model is performed. For both approaches, an application to a specific material model – barodesy – is shown. For the simulated standard laboratory tests, both approaches show almost the same results and are an improvement compared with the original model behaviour. Both approaches also work for elastoplastic material models, which is demonstrated for the MCCM. The original intergranular strain concept is a well‐established concept to improve small‐strain behaviour in hypoplastic models, but it still also has its shortcomings. However, the possibility to apply the intergranular strain concept to more constitutive models will make it available for a wider range of constitutive modellers and may also help to develop improvements for the concept.

## AUTHOR CONTRIBUTIONS

M.B, G.M., and W.F developed the concept of the article. M.B. and W.F. developed the IEM, supported by A.O.; D.M. and M.B. worked out the EEM approach. A.O. delivered mathematical foundations. M.B. performed the calculations and analyses. M.B. mainly wrote the article with input from all authors.

## APPENDIX 1 EQUATIONS OF BARODESY

In this appendix, the equations of barodesy for clay^4^ are summarized:

(A1) $$\overset{\circ }{T}=h\cdot \left(fR^{0}+gT^{0}\right)\|D\|,\;$$

(A2) $$R=-\exp(\alpha D^{0})\;\;\text{with}\;\;\alpha =\frac{\ln K}{\sqrt{3/2-{\left(\text{tr}\;D^{0}\right)}^{2}/2}},$$

(A3) $$\;K=1-\frac{1}{1+c_{1}{\left(m-c_{2}\right)}^{2}}\;\;\text{with}\;\;m=\frac{-3\text{tr}\;D^{0}}{\sqrt{6-2{\left(\text{tr}\;D^{0}\right)}^{2}}},$$

(A4) $$h=c_{3}\|T{\|}^{c_{4}},$$

(A5) $$f=c_{6}\beta \;\text{tr}\;D^{0}-\frac{1}{2},\;$$

(A6) $$g=(1-c_{6})\beta \;\text{tr}\;D^{0}+{\left(\frac{1+e}{1+e_{c}}\right)}^{c_{5}}-\frac{1}{2},\;$$

(A7) $$e_{c}=\exp\left(N-\lambda^{*}\ln\frac{-2\;\text{tr}\;T}{3\sigma^{*}}\right)-1,\;$$

(A8) $$\beta =-\frac{1}{c_{3}\Lambda }+\frac{1}{\sqrt{3}}2^{c_{5}\lambda^{*}}-\frac{1}{\sqrt{3}},\;$$

(A9) $$\Lambda =-\frac{\lambda^{*}-\kappa^{*}}{2\sqrt{3}}\text{tr}\;D^{0}+\frac{\lambda^{*}+\kappa^{*}}{2}.\;$$

The constants $c_{1}$ to $c_{6}$ can be determined on the basis of the critical state soil mechanics parameters $\varphi_{c}$, N, $\lambda^{*}$, and $\kappa^{*}$.^4^

## References

[1] Kolymbas D. A generalized hypoelastic constitutive law. In: Publications Committee of XI. ICSMFE, eds., Int. Conf. Soil Mechanics and Foundation Engineering Balkema; 1985; San Francisco: 2626.

[2] Kolymbas D . An outline of hypoplasticity. Arch of Appl Mech. 1991;63(3):143‐151.

[3] Kolymbas D . Barodesy: a new hypoplastic approach. Inter J Num Anal Met in Geomech. 2012;36(9):1220‐1240.

[4] Medicus G , Fellin W . An improved version of barodesy for clay. Acta Geotech. 2017;12(2):365‐376.

[5] Herle I . On basic features of constitutive models for geomaterials. J of Theo and Appl Mech. 2008;38(1‐2):61‐80.

[6] Benz T. Small‐strain stiffness of soils and its numerical consequences. Mitteilung des Instituts für Geotechnik, Stuttgart: Institut für Geotechnik 2007.

[7] Niemunis A , Herle I . Hypoplastic model for cohesionless soils with elastic strain range. Mech of Cohes frict Mat. 1997;2(4):279‐299.

[8] Wegener D , Herle I . Prediction of permanent soil deformations due to cyclic shearing with a hypoplastic constitutive model. geotechnik. 2014;37(2):113‐122.

[9] Mašín D . Clay hypoplasticity model including stiffness anisotropy. Géotechnique. 2014;64(3):232‐238.

[10] Fuentes W , Triantafyllidis T . ISA model: a constitutive model for soils with yield surface in the intergranular strain space. Inter J Num Anal Met in Geomech. 2015;39(11):1235‐1254.

[11] Niemunis A. Extended hypoplastic models for soils. 34. Inst. für Grundbau und Bodenmechanik. 2003.

[12] Stutz HH , Wuttke F . Hypoplastic modeling of soil‐structure interfaces in offshore applications. J of Zhejiang Univ‐SCIENCE A. 2018;19(8):624‐637.

[13] von Wolffersdorff P–A , Schwab R . The Uelzen I Lock—hypoplastic finite‐element analysis of cyclic loading. Bautechnik. 2009;86(S1):64‐73.

[14] Mašín D , Herle I . Numerical analyses of a tunnel in London clay using different constitutive models. In: Bakker K, Bezuijen A, Broere W, Kwast E. , eds., *5th International Symposium TC28 Geotechnical Aspects of Underground Construction in Soft Ground*; 2005: 2–4.

[15] Wichtmann T , Fuentes W , Triantafyllidis T . Inspection of three sophisticated constitutive models based on monotonic and cyclic tests on fine sand: Hypoplasticity vs. Sanisand vs. ISA. Soil Dyn and Earthquake Eng. 2019;124:172‐183.

[16] von Wolffersdorff P‐A . A hypoplastic relation for granular materials with a predefined limit state surface. Mech of Cohes frict Mat. 1996;1(3):251‐271.

[17] Wu W . Hypoplastizität als mathematisches Modell zum mechanischen Verhalten granularer Stoffe. Veröffentlichungen des Institutes für Bodenmechanik und Felsmcheanik der Universität Fridericiana in Karlsruhe, Karlsruhe: Institut für Bodenmechanik und Felsmechanik 1992.

[18] Gudehus G . A comprehensive constitutive equation for granular materials. Soils and Found. 1996;36(1):1‐12.

[19] Kolymbas D . Sand as an archetypical natural solid In: Kolymbas D, Viggiani G, eds. Mechanics of Natural Solids. Berlin: Springer; 2009:1‐26.

[20] Fellin W , Ostermann A . The critical state behaviour of barodesy compared with the Matsuoka–Nakai failure criterion. Inter J Num Anal Met in Geomech. 2013;37(3):299‐308.

[21] Mašín D . Clay hypoplasticity with explicitly defined asymptotic states. Acta Geotech. 2013;8(5):481‐496.

[22] Costanzo D , Viggiani G , Tamagnini C . Directional response of a reconstituted fine‐grained soil—part I: experimental investigation. Inter J Num Anal Met in Geomech. 2006;30(13):1283‐1301.

[23] Hettler A , Danne S . Strain response envelopes for low‐cycle loading processes In: Delage P, Desrues R, Puech A, Schlosser F, eds. Proceedings of the 18th International Conference on Soil Mechanics and Geotechnical Engineering. Paris; 2013:1491‐1494.

[24] Mašín D . Laboratory and numerical modelling of natural clays. MPhil thesis. City University, London, 2004.

[25] Hardin B.O , Black WL . Vibration modulus of normally consolidated clay. J of the Soil Mech and Found Div. 1969;94(2):353‐370.

[26] Biarez J , Hicher PY . Elementary Mechanics of Soil Behaviour: Saturated Remoulded Soils. Rotterdam: Balkema; 1994.

[27] Mašín D . A hypoplastic constitutive model for clays. Inter J Num Anal Met in Geomech. 2005;29(4):311‐336.

[28] Roscoe K , Burland J . On the generalized stress‐strain behaviour of wet clay In: Heyman J, Leckie F, eds. Engineering Plasticity. Cambridge: Cambridge University Press; 1968:535‐609.

[29] Mašín D , Rott J . Small strain stiffness anisotropy of natural sedimentary clays: review and a model. Acta Geotech. 2013;9(2):299‐312.

[30] Fuentes W , Triantafyllidis T , Lascarro C . Evaluating the performance of an ISA‐hypoplasticity constitutive model on problems with repetitive loading In: Triantafyllidis T, ed. Holistic Simulation of Geotechnical Installation Processes: Theoretical Results and Applications. Cham: Springer International Publishing; 2017:341‐362.

